# Preparations and Characterizations of Luminescent Two Dimensional Organic-inorganic Perovskite Semiconductors

**DOI:** 10.3390/ma3053385

**Published:** 2010-05-25

**Authors:** Sanjun Zhang, Pierre Audebert, Yi Wei, Antoine Al Choueiry, Gaëtan Lanty, Antoine Bréhier, Laurent Galmiche, Gilles Clavier, Cédric Boissière, Jean-Sébastien Lauret, Emmanuelle Deleporte

**Affiliations:** 1 Laboratoire de Photonique Quantique et Moléculaire, Ecole Normale Supérieure de Cachan, 61 avenue du Président Wilson, 94235 Cachan, France; 2 Laboratoire de Photophysique et Photochimie Supramoléculaires et Macromoléculaires, Ecole Normale Supérieure de Cachan, 61 avenue du Président Wilson, 94235 Cachan, France; E-Mail: pierre.audebert@ppsm.ens-cachan.fr (P.A.); 3 State Key Laboratory of Precision Spectroscopy, Department of Physics, East China Normal University, No.3663, North Zhongshan Road, Shanghai 200062, P. R. China; E-Mail: sjzhang@phy.ecnu.edu.cn (S.Z.); 4 Laboratoire de Chimie de la Matière Condensée, UMR 7574, 4 place Jussieu, T54 E5 55-54, 75252 Paris Cedex 05, France

**Keywords:** organic-inorganic perovskites, hybrid materials, multi-quantum wells, nanoparticles, spin coating, X-ray diffraction, absorption spectrum, photoluminescence

## Abstract

This article reviews the synthesis, structural and optical characterizations of some novel luminescent two dimensional organic-inorganic perovskite (2DOIP) semiconductors. These 2DOIP semiconductors show a self-assembled nano-layered structure, having the electronic structure of multi-quantum wells. 2DOIP thin layers and nanoparticles have been prepared through different methods. The structures of the 2DOIP semiconductors are characterized by atomic force microscopy and X-ray diffraction. The optical properties of the 2DOIP semiconductors are characterized from absorption and photoluminescence spectra measured at room and low temperatures. Influences of different components, in particular the organic parts, on the structural and optical properties of the 2DOIP semiconductors are discussed.

## 1. Introduction

Two dimensional organic-inorganic perovskites (2DOIPs) are a wide class of materials, with general chemical structures (R-NH${}_{3}$)${}_{2}$MX${}_{4}$ or (H${}_{3}$N-R-NH${}_{3}$)MX${}_{4}$, where R is an organic group, M a divalent metal (such as, Pb${}^{2+}$, Sn${}^{2+}$, Ge${}^{2+}$, Cu${}^{2+}$, Ni${}^{2+}$, Mn${}^{2+}$, Fe${}^{2+}$, Co${}^{2+}$, Eu${}^{2+}$), and X a halogen (Cl${}^{-}$, Br${}^{-}$, I${}^{-}$). Some of the 2DOIPs present interesting optical properties, such as photoluminescence (PL) and electroluminescence (EL) at room temperature. The luminescent 2DOIPs are potential materials for realizing laser diodes (LD) and organic-inorganic light emitting diodes (OILED) [1,2,3,4,5,6]. Another important potential application of the 2DOIPs is the realization of polariton lasers, for which the strong coupling regime between the excitons of the active medium and the photon modes of a photonic structure is needed. We have recently demonstrated the strong coupling of the excitons in 2DOIPs with the photon modes in two different configurations: a Fabry-Perot microcavity [7,8,9], and surface plasmons [10,11]. Moreover, it can be found in the literature that the 2DOIPs are also potential materials for nonlinear optical devices [12].

**Figure 1:**
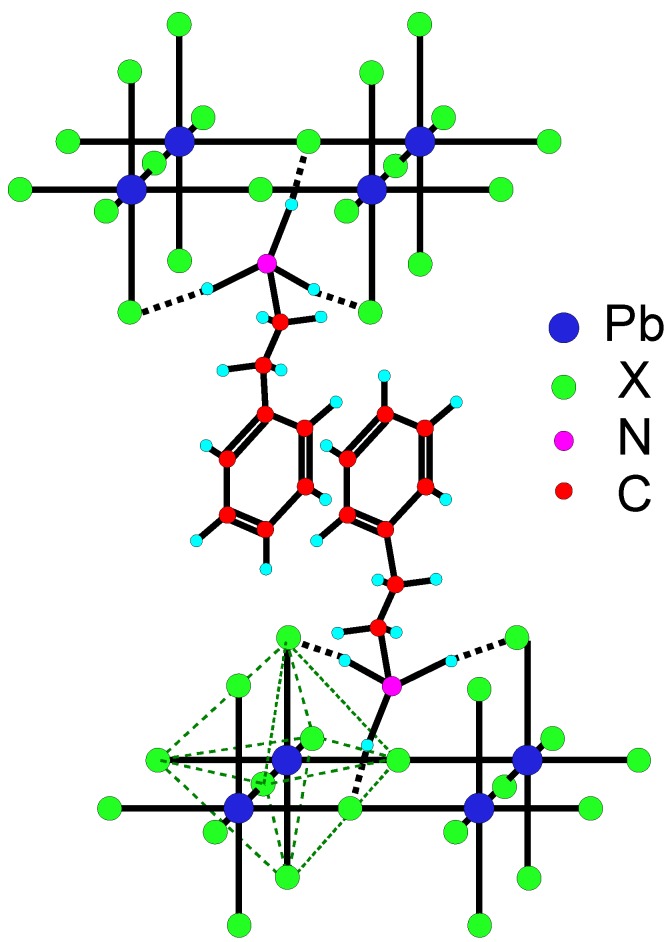
Sketch of the 3D structure of the (C${}_{6}$H${}_{5}$C${}_{2}$H${}_{4}$NH${}_{3}$)${}_{2}$PbX${}_{4}$ (X=I, Br, or Cl) 2DOIPs.

Figure 1 illustrates the structure of the (C${}_{6}$H${}_{5}$C${}_{2}$H${}_{4}$NH${}_{3}$)${}_{2}$PbX${}_{4}$ (X=I, Br, or Cl) 2DOIPs. Since 2-phenylethanamine is frequently used as the organic group of the 2DOIPs in this paper, we note (C${}_{6}$H${}_{5}$C${}_{2}$H${}_{4}$NH${}_{3}^{+}$)${}_{2}$ as PhE hereafter for convenience. For example, (C${}_{6}$H${}_{5}$C${}_{2}$H${}_{4}$NH${}_{3}$)${}_{2}$PbI${}_{4}$ will be referred as PhE-PbI${}_{4}$. As illustrated in Figure 1, each metal atom and its 6 neighbouring halide atoms form a MX${}_{6}^{4-}$ octahedron. The octahedrons constitute an inorganic layer by sharing the halide atoms with its neighboring octahedrons in 2 dimensions [13,14,15,16,17]. Organic ammonium salts can bind to the inorganic layers and form an intercalated 2D organic-inorganic layers structure.

Energy gap in an organic material is defined as the energy difference between the highest occupied molecular orbital (HOMO) and lowest unoccupied molecular orbital (LUMO) bands. Since the energy gap of the organic layers is usually much larger than that of the inorganic layers, the electronic structure of 2DOIPs can thus be regarded as a multi-quantum-well, consisting in inorganic wells and organic barriers. The optical properties of the 2DOIPs are generally decided by the inorganic layers. Due to the large variety of MX${}_{2}$ semiconductors, the principle optical properties of 2DOIPs may be changed by adopting different MX${}_{2}$ semiconductors. Besides, the optical properties of 2DOIPs can also be tailored by the organic groups. The large flexibility in the choice of the organic and inorganic components has a positive consequence: the 2DOIPs can be artificially designed to afford many interesting properties. We will concentrate on the optical properties of 2DOIPs in this paper. The techniques used to prepare the 2DOIPs and to characterize the properties of these 2DOIPs will be introduced. The influence of the organic and inorganic components will be studied, leading to a discussion about how to select the organic components to improve the optical properties of the 2DOIPs.

## 2. Preparation of the 2DOIPs

The 2DOIP crystals exist as bulks, thin films, and nanoparticles [18,19,20,21]. In each case, various preparation methods have been developed. Bulk crystals can be prepared by slow cooling the saturated solution [18,19]. The preparation of 2DOIP crystals are generally performed in two steps if necessary. The first step is to transform amine to ammonium salt by reacting with hydrogen halide (HX),

(1) $$R-{\mathrm{NH}}_{2}+\mathrm{HX}\Rightarrow R-{\mathrm{NH}}_{2}\cdot \mathrm{HX}$$

The second step is to dissolve the $R-{\mathrm{NH}}_{2}\cdot \mathrm{HX}$ and semiconductors MX${}_{2}$ in solvent and to prepare the 2DOIP crystals from solution,

(2) $$2\left(R-{\mathrm{NH}}_{2}\cdot \mathrm{HX}\right)+{\mathrm{MX}}_{2}\Rightarrow {\left(R-{\mathrm{NH}}_{3}\right)}_{2}{\mathrm{MX}}_{4}$$

The two step procedure is described in details in paragraphs 2.1 and 2.2. The nanoparticles are generally prepared in solution by evaporation of the solvents [21], we have prepared them by a spray-drying method which we will describe in paragraph 2.3.

### 2.1. Ammonium salts

Amines (monoamine R-NH${}_{2}$ or diamine H${}_{2}$N-R-NH${}_{2}$) can be transformed to ammonium salts by reacting with hydrogen halide (HX, X = Cl, Br, and I). This reaction is described by Equation 1. Gaseous HX is generated in a closed bottle as described in the following. Gaseous HCl can be obtained by dropping concentrated H${}_{2}$SO${}_{4}$ solution gingerly on NaCl powder, this reaction is friendly. However, gaseous HBr or HI are obtained by dropping concentrated HI or HBr acid gingerly on P${}_{2}$O${}_{5}$ powder due to the oxidation of concentrated sulfuric acid to HBr and HI. The dehydration process of P${}_{2}$O${}_{5}$ to HBr and HI solution is very violent and exothermic, so special attention should be given to this reaction and it should be cooled by ice-water. Gaseous HX is then guided through a tube to a closed bottle with diethyl ether containing R-NH${}_{2}$. HX reacts with R-NH${}_{2}$ in diethyl ether under agitation as described by Equation 1. R-NH${}_{2}$ is usually soluble in diethyl ether, however the solubility of R-NH${}_{2}\cdot$HX is usually very low in diethyl ether. Thus R-NH${}_{2}\cdot$HX salts precipitate after this reaction. The unreacted gaseous HX is guided to another bottle with NaOH in water solution and is neutralized. Two additional security bottles are strongly suggested to be introduced into the reaction setup (one between the bottle of gaseous HX generation and the bottle of $R-{\mathrm{NH}}_{2}\cdot \mathrm{HX}$ salts generation reaction, and the other between the bottle of $R-{\mathrm{NH}}_{2}\cdot \mathrm{HX}$ salts generation reaction and the bottle of the neutralization reaction) to avoid the backward flow caused by the variation of pressure. The $R-{\mathrm{NH}}_{2}\cdot \mathrm{HX}$ salts are then rinsed and filtered two times by diethyl ether and one time by n-pentane to remove the unreacted HX or R-NH${}_{2}$. The $R-{\mathrm{NH}}_{2}\cdot \mathrm{HX}$ salts are stored in dry box for several days to remove the residual water before usage. $R-{\mathrm{NH}}_{2}\cdot \mathrm{HCl}$ and $R-{\mathrm{NH}}_{2}\cdot \mathrm{HBr}$ are generally stable under oxygen in dry environment. However, $R-{\mathrm{NH}}_{2}\cdot \mathrm{HI}$ may be slowly oxidized if exposing in air.

### 2.2. Thin films

Ammonium salts R-NH${}_{2}$HX and semiconductors MX${}_{2}$ are dissolved in a solvent in stoichiometry of 2:1, and then 2DOIP crystals may be obtained by evaporation of solvent. The formation of 2DOIP crystals is a self-organization process as described by Equation 2. The choice of the solvent is an important issue for the solution based preparation method of 2DOIP crystals. A solvent which is able to dissolve both R-NH${}_{2}$·HX and semiconductors MX${}_{2}$ is needed for this preparation. Water is generally able to dissolve the ammonium salts R-NH${}_{2}$·HX, but not the MX${}_{2}$ semiconductors. Various solvents may be potentially convenient for 2DOIP, such as, *n*-methyl-2-pyrrolidone, glycol, methanol, ethanol, acetone, and isopropanol [18]. It has been found that the solubility increases as the dielectric constant of the solvent increases [22]. Thus, a solvent with high dielectric constant, dimethylformamide (DMF), is usually used in our experiments, and it has been proved to afford enough solubility to most 2DOIP crystals [20]. If necessary, dimethyl sulfoxide (DMSO) is also used in our experiments, which can normally afford higher solubility than DMF to the 2DOIP materials. More than one ammonium salts R-NH${}_{2}$·HX and semiconductors MX${}_{2}$ can be dissolved in the same solvent to prepare some hybrid materials with interesting properties. For examples, C${}_{6}$H${}_{5}$C${}_{2}$H${}_{4}$NH${}_{2}\cdot$HCl, C${}_{6}$H${}_{5}$C${}_{2}$H${}_{4}$NH${}_{2}\cdot$HBr, PbCl${}_{2}$ and PbBr${}_{2}$ can be dissolved in DMF in stoichiometry to prepare excitonic energy adjustable PhE-PbBr${}_{x}$Cl${}_{4-x}$ semiconductors, whose emission energy depends on *x*, as it will be described in details in paragraph 4.1.

#### 2.2.1. Spin-coating

Spin-coating is a simple method to prepare thin films. The process of spin-coating is complex, and it is thus difficult to be modelize it. The film thickness *h* is usually described by an empirical formula [9,23,24,25],

(3) $$h=\frac{C}{\sqrt{t}}\omega^{-x}$$

where ω and *t* are the spin speed and duration, respectively. *C* is an experimentally determined constant, which depends on the evaporation rate, viscosity and density of the solution. *x* is also an experimentally determined parameter, which is related to the evaporation rate of the solution. x≈1/2 for most solvents. x≈2/3 when the evaporation rate is independent of ω, and x≈1 for slow evaporation solvent. The solvent containing R-NH${}_{2}$·HX and MX${}_{2}$ is first spin-coated on the substrate. Then 2DOIP crystals may be obtained by evaporation of solvent. It is worth noting that the 2DOIP crystals only form from its molecular components at the late stage of solvent evaporation, when the liquid layer has become stagnant. In the absorption spectra of 2DOIP crystals, a sharp peak appears at room temperature, which is characteristic of the formation of 2DOIP crystal structure (see paragraph 3.2). By carefully selecting the parameters, homogeneous 2DOIP films having a thickness from 10 to 100 nm can be easily obtained. The spin-coated 2DOIP films are very reproducible, and are appropriate to make devices [25].

An important issue for preparing homogeneous 2DOIP films is the surface effect related to the substrate. The surface charge of substrate can drastically change the properties of the 2DOIP films. Prior to preparing 2DOIP films on glass or quartz slides, we first cleaned the surface subsequently by acetone, ethanol, and propanol in ultrasonic bath, each step lasted for 15 minutes [20]. After that, the quartz or glass slides were immersed in 1 mol L${}^{-1}$ KOH in ethanol solution for 15 min for modifying the surface charge [17]. Then the slides were rinsed by distilled water and dried by nitrogen flow. The homogeneity of 2DOIP films prepared on KOH treated surface is better than on untreated surface. Besides the simple treatment of surface by KOH, another advanced but more complicated method, namely self-assembled monolayer (SAM), can also be used to protect the surface, and/or to improve the film properties of 2DOIP films on various substrates. The molecule used to prepare SAMs can generally be divided into three parts: head group, backbone and end group. The head group is a specific linker to each type of substrate, for example, S or N atoms for clean metals, and Si or P for hydroxylated and oxidized surfaces [26]. The concentration of SAM molecules in solution is generally very low (10${}^{-4}∼$10${}^{-3}$ mol L${}^{-1}$). After dipping the substrate in a solution containing SAM molecules for a long time (for example an overnight), a highly ordered molecule monolayer may form on substrate due to self-assembling [27]. One advantage of the SAMs is that various functional end groups can be selected to arbitrarily modify the surface properties. Molecules whose end group has affinity to the interesting materials are usually selected to prepare SAMs to improve the adhesion of these materials on substrate. We have experimentally observed that the spin coated PhE-PbI${}_{4}$ films on 4-aminobenzenethiol SAM modified metal surface are more homogeneous than on unmodified surface probably due to the hydrogen bonds between the amino groups and the perovskite.

#### 2.2.2. Thermal ablation

2DOIP films prepared through thermal ablation have also been reported [28,29,30]. Thermal ablation preparation of 2DOIP is interesting even if it is more complicated than spin-coating. The main reason is that the thermal ablation technique of 2DOIPs is compatible with the thermal ablation of many other materials, thus multi-layered structures that are widely used in LDs, LEDs, and micro-cavities can be achieved at once with multi-source thermal ablation. Various organic salts (C${}_{6}$H${}_{5}$C${}_{2}$H${}_{4}$NH${}_{2}\cdot$HI, and C${}_{n}$H${}_{2n+1}$NH${}_{2}\cdot$HI, (*n*=4, 6, 10)) and PbI${}_{2}$ were simultaneously deposited by dual-source vapor deposition in ref.[28]. These thermally deposited films were uniform, and showed strong excitonic absorption. Using separate sources for organic and inorganic components in the dual-source thermal evaporation makes it possible to apply different powers and temperature conditions for the two components. However the relative evaporation rate of the two components should be carefully adjusted to reach a balance. Mitzi *et al.* have developed a single source thermal ablation method [29]. Organic-inorganic perovskites in the form of a crystal, a powder, or a concentrated solution can be used in the single source thermal ablation method. After vacuum is established, a very large current was applied to the heater to evaporate the organic-inorganic perovskites. The heater temperature reached approximately 1000 °C in 1-2 s. Numerous 2DOIPs, including PhE-PbI${}_{4}$, PhE-PbBr${}_{4}$, and PhE-SnI${}_{4}$, have been successfully deposited by this single source thermal ablation technique. X-ray diffraction and absorption measurements showed that these examples have good self-organized structure [29].

### 2.3. Nanoparticles

**Figure 2:**
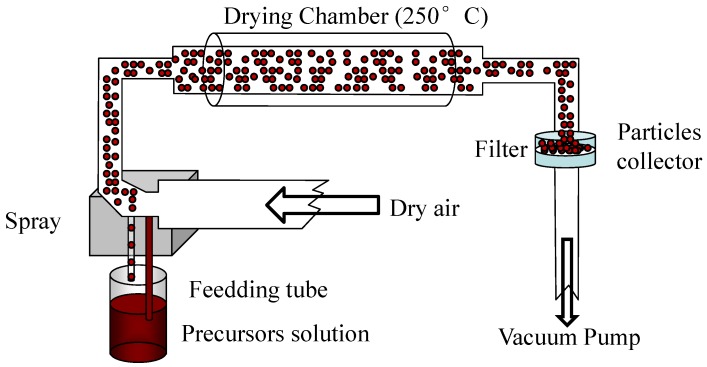
Sketch for the preparation of organic-inorganic perovskite nanoparticles through spray-drying.

We have realized luminescent 2DOIP nanoparticles by a spray-drying (or nebulization/lyophilization) method [21]. Similarly to the spin-coating, the R-NH${}_{2}$·HX ammonium salts and PbBr${}_{2}$ or PbI${}_{2}$ semiconductors were firstly dissolved in DMF solvent in stoichiometry of 2:1. The preparation method is sketched in Figure 2. The experimental spray drier, shown in Figure 2, is composed of an aerosol generator and an evaporation chamber which is settled in an oven maintaining at 250 °C. Droplets with initial mean diameter of 0.35 μm were carried by dry air (3 L·min${}^{-1}$) from the aerosol generator to the evaporation chamber. Dried particles were collected onto a 0.2 μm cutoff Teflon filter and were stored at ambient temperature. TEM measurements show that these particles are spherical and their sizes are of the order of 50 to 500 nm [21].

## 3. Characterizations

### 3.1. Structural characterizations

#### 3.1.1. Atomic force microscopy

**Figure 3:**
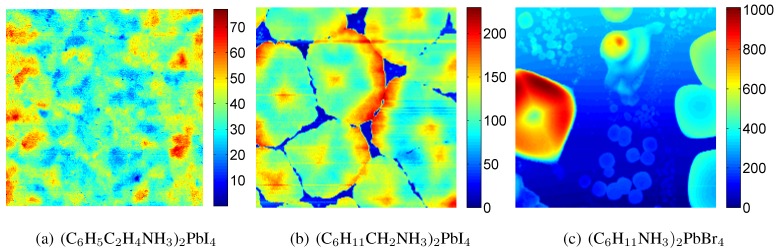
Atomic force microscopy images of 3 different 2DOIPs self-organized on glass substrate. The chemical formula of each 2DOIP is shown in the caption of (a), (b), and (c), respectively. The lateral size of each image is 20×20 μm${}^{2}$. The color coding of the vertical scale of each image is displayed in the respective sidebar in nanometer. The scales in height of the images are 75 nm (a), 230 nm (b), and 1000 nm (c), respectively. Please refer to ref. [20] for the details of film preparation.

Atomic force microscopy (AFM) is a powerful tool for determining the surface topography with nanometer scale resolution [31,32,33]. Depending on the organic and inorganic components of 2DOIPs, their self-organized structure on substrate may be very different. The self-organized structure can be comparatively studied by AFM. Figure 3 presents the topography of three different 2DOIPs on glass substrate. The topographies of these films are very different. The maxima in height are 74 nm (a), 230 nm (b), and 998 nm (c), respectively. It can be seen that the surface of Figure 3(a) is fully covered by (C${}_{6}$H${}_{5}$C${}_{2}$H${}_{4}$NH${}_{3}$)${}_{2}$PbI${}_{4}$. If we define the height less than 10% of the maximum of topography is not covered by 2DOIP, the surface coverage can be found to be 97% and 76% for Figure 3(b) and Figure 3(c), respectively. The difference is related to the nature of the organic components [20]. In the bi-organic layer 2DOIPs, the organic groups interact with each other to form a self-organized structure. For example, the organic component is 2-phenylethanamine in PhE-PbI${}_{4}$, it is known that there exists π-π interactions between adjacent phenyl groups, leading to a good film quality for PhE-PbI${}_{4}$. The organic component is cyclohexylmethanamine in (C${}_{6}$H${}_{11}$CH${}_{2}$NH${}_{3}$)${}_{2}$PbI${}_{4}$, the interaction between the organic components consists in weak van der Waals forces, thus the (C${}_{6}$H${}_{11}$CH${}_{2}$NH${}_{3}$)${}_{2}$PbI${}_{4}$ film is discontinuous and more rough. The organic component is cyclohexanamine in (C${}_{6}$H${}_{11}$NH${}_{3}{)}_{2}$PbBr${}_{4}$. The ammonium unit penetrates into the inorganic sheets when it binds to the metal halide octahedron through hydrogen bonding, a spacer is needed when the organic group is too large to fit into the hole within the inorganic sheet. From geometric calculations it is possible to see that the distance between the ammonium unit and cyclohexane is too short, the consequence is an increase of the roughness of the (C${}_{6}$H${}_{11}$NH${}_{3}{)}_{2}$PbBr${}_{4}$ thin films [20].

#### 3.1.2. X-ray diffraction

**Figure 4:**
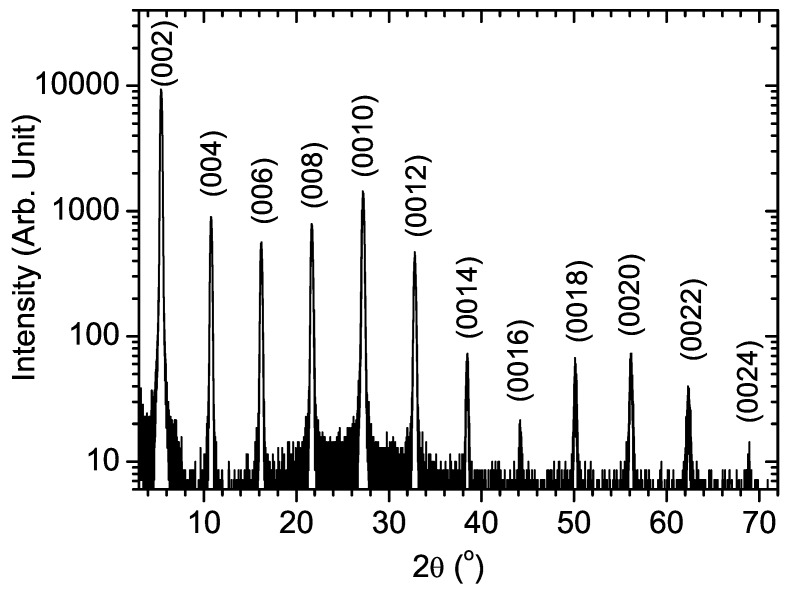
X-ray diffraction patterns of a spin-coated 50-nm-thick PhE-PbI${}_{4}$ layer.

X-ray diffraction (XRD) is a frequently used method to characterize the ordering within the self-organized 2DOIPs [34,35]. Figure 4 presents XRD pattern measured on a spin-coated 50-nm-thick PhE-PbI${}_{4}$ film. Numerous diffraction orders from (002) to (0024) can be observed, which proves the high crystallinity of the PhE-PbI${}_{4}$ film. A period of 16.4 Å can be accurately estimated from the patterns of Figure 4. Please note this period is not the unit cell length, which should be the double (32.8 Å). The lattice parameter of the PbI${}_{6}^{4-}$ octahedrons has been measured to be 6.325 Å [17], thus the organic part can be deduced to be 10 Å along the growth direction. XRD technique can also be used to characterize the 2DOIP nanopaticles [21]. The XRD patterns obtained from PhE-PbI${}_{4}$ films and nanoparticles are quite comparable [21]. However X-ray crystallographic measurement is needed to know the more detailed in-plan structures of the 2DOIP crystals [36].

### 3.2. Optical Characterizations

#### 3.2.1. Absorption spectrum at room temperature

**Figure 5:**
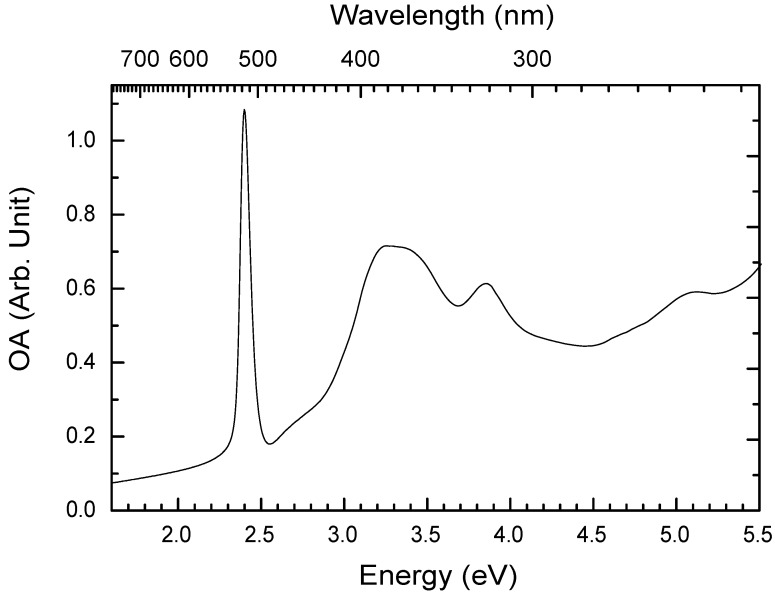
Optical absorbance (OA) spectrum of a 50-nm-thick PhE-PbI${}_{4}$ film measured at room temperature.

Figure 5 presents the UV-Vis optical absorbance (OA) spectrum of a spin-coated 50-nm-thick PhE-PbI${}_{4}$ film measured at room temperature. The PhE-PbI${}_{4}$ film was prepared by spin-coating 0.1 mol L${}^{-1}$ perovskites in DMF on quartz slide at 1500 rpm for 30 seconds. The OA (also called optical density) is defined as

(4) $$\mathrm{OA}\left(\lambda \right)=-\log_{10}\left(I/I_{0}\right)$$

where $I_{0}$ and *I* are the intensities of a monochromatic light of wavelength (λ) before and after it passes through the sample. A sharp absorption peak is seen at 2.40 eV in Figure 5. This peak does not exist in a solution. This absorption peak is attributed to the excitonic absorption of the thin film. In the 2DOIPs, the excitons are highly located in the inorganic sheets because the energy gap of the organic sheets is usually much larger than that of the inorganic sheets. The absorption peak at lowest energy (2.4 eV) is attributed to the electronic transition from a mixture of Pb(6s)-I(5p) states (valence band) to Pb(6p) state (conduction band) for PbI${}_{2}$ based 2DOIPs [37]. The absorption peaks at higher energy in Figure 5 are thought to originate from 6s-6p transitions in Pb${}^{2+}$[38]. Interaction between Pb (6p) and I (5p) determines the crystal field of 2DOIPs. Crystal field and spin-orbit effects leave the degeneracy of the Pb 6s-6p transition and are responsible of the multiple absorption bands shown in Figure 5 [38,39]. By supposing the crystal structure to be a square lattice of PbI${}_{6}^{4-}$ and using the tight binding band structure model, it has been found that the direct transition in the corner of a square Brillouin zone (M point) brings the absorption peak at 2.4 eV, and the interband transitions at the M point give the absorption bands at 3.2 and 3.8 eV [39].

#### 3.2.2. Ellipsometry

**Figure 6:**
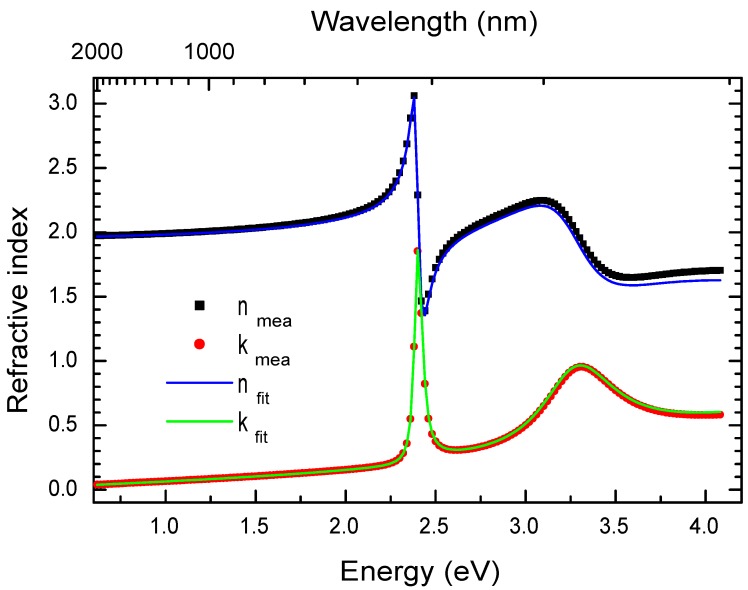
Refractive index (n+ik) of PhE-PbI${}_{4}$ film measured by ellipsometry. The 200-nm-thick PhE-PbI${}_{4}$ film was spin-coated on silicon substrate. The black squares and red dots represent the real ($n_{mea}$) and imaginary ($k_{mea}$) parts of the experimentally measured refractive index, respectively. The blue and green solid lines present the fit to the experimental data with the Equation 7 and the parameters reported in Table 1.

Ellipsometry is a technique to measure the complex refractive index (n+ik) of materials. Figure 6 shows the *n* and *k* components of the refractive index of a PhE-PbI${}_{4}$ film measured by an ellipsometer. The PhE-PbI${}_{4}$ film was deposited on a silicon substrate by spin-coating, the thickness of the film was measured to be 200 nm by a profilemeter. Several peaks have been seen in the absorption spectrum, we modeled each absorption peak by a Lorentz oscillator. The contribution of each Lorentz oscillator to the dielectric constant is:

(5) $$\epsilon \left(E\right)=\frac{A}{E_{0}^{2}-E^{2}-j\hbar \gamma E}$$

where, $E_{0}$ (in eV) is the energy position of the absorption peak, γ (in eV) is the width of the absorption peak, and *A* (in eV${}^{2}$) is related to the oscillator strength $f_{osc}$:

(6) $$A=\frac{\hbar^{2}}{\epsilon_{0}m_{0}}\frac{f_{osc}}{L_{tot}}$$

where, $L_{tot}$ is the thickness of the film.

We modeled the dielectric constant of PhE-PbI${}_{4}$ by three Lorentz oscillators:

(7) $$\epsilon =\epsilon_{\infty }+\epsilon_{1}+\epsilon_{2}+\epsilon_{3}$$

where, $\epsilon_{\infty }$ is a constant, $\epsilon_{1}$ and $\epsilon_{2}$ are related to the peaks at 2.4 and 3.2 eV respectively. $\epsilon_{3}$ is a large peak which models the continuous absorption [40]. Figure 6 shows the real ($n_{mea}$) and imaginary ($k_{mea}$) parts of the experimentally measured refractive index ($n+ik=\sqrt{\epsilon }$) of PhE-PbI${}_{4}$ (squares and dots, respectively). The experimental curves can be fitted by the Equation 7. The optimum parameters to fit the experimental PhE-PbI${}_{4}$ refractive index curves with Equation 7 are reported in Table 1.

The oscillator strength $f_{osc}$ can be obtained from Equation 6, knowing *A* and $L_{tot}$ values. We obtain $f_{osc}$ = 13× 10${}^{15}$ cm${}^{-2}$ for PhE-PbI${}_{4}$ films. Oscillator strength per quantum-well $f_{qw}$ = 9× 10${}^{13}$ cm${}^{-2}$ can be deduced by dividing the total $f_{osc}$ with the number of quantum wells N${}_{qw}$ = 150. This is one order of magnitude higher than in conventional inorganic quantum wells such as InGaAs structure [41]. In order to be compared to classical oscillators and a vast number of other atomic, molecular and solid state data, one can divide the oscillator strength per quantum-well value by the surface density of PbI${}_{4}$ groups, one then finds 0.36 a fairly high value comparable to that in luminescent dyes.

#### 3.2.3. Photoluminescence spectrum at room temperature

**Figure 7:**
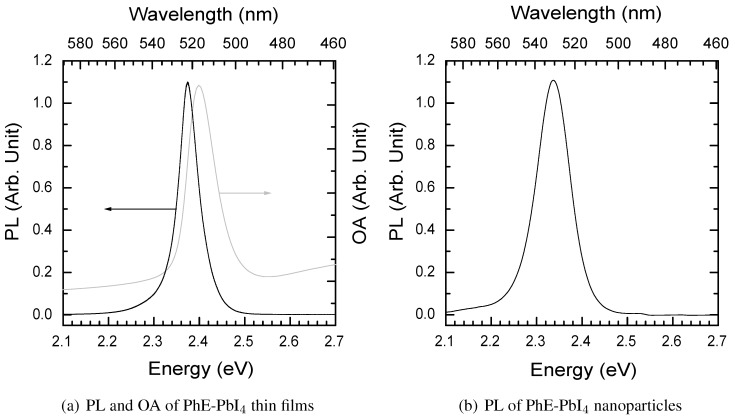
Photoluminescence (PL) spectra of a 50-nm-thick thin film (a), and nanoparticles (b) of PhE-PbI${}_{4}$. The PL was excited with the 325 nm line of a HeCd laser. OA spectrum of PhE-PbI${}_{4}$ film is also presented in figure (a) in gray for comparison.

**Figure 8:**
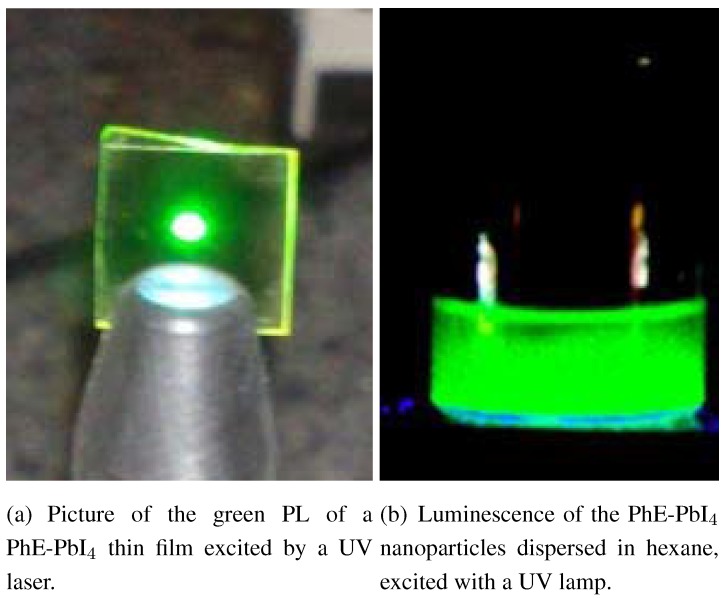
Photoluminescence pictures of a 50-nm-thick thin PhE-PbI${}_{4}$ film (a), and PhE-PbI${}_{4}$ nanoparticles (b).

For the 2DOIPs that present a characteristic absorption peak related to the 2D arrangement, some of them may have very efficient luminescence (such as the (C${}_{6}$H${}_{11}$CH${}_{2}$NH${}_{3}{)}_{2}$PbBr${}_{4}$ in ref [20]), but some of them does not have luminescence (such as the (C${}_{8}$H${}_{15}$NH${}_{3}{)}_{2}$PbBr${}_{4}$ in ref [20]). Photoluminescence (PL) spectrum gives a direct characterization of the luminescence ability of the 2DOIPs. Figure 7 shows the PL spectra of a 50-nm-thick PhE-PbI${}_{4}$ thin films (a), and of PhE-PbI${}_{4}$ nanoparticles (b) measured at room temperature. The PL was excited with the 325 nm line of a HeCd laser, was collected by an optical lens and was coupled into a spectrometer. Figure 8 shows pictures of the photoluminescence of a thin film and of nanoparticles. The PL of the PhE-PbI${}_{4}$ thin film consists in a bright green light centered at 2.375 eV ($\lambda_{PL}$ = 522 nm ). The full width at half maximum (FWHM) of the PhE-PbI${}_{4}$ thin film is 51 meV, which is much narrower than that of some luminescent organic polymers and laser dyes. OA spectrum of the PhE-PbI${}_{4}$ film is also shown in Figure 7(a) in gray for comparison. Stokes shift is defined as the energy difference between absorption and PL maxima. The Stokes shift in 2DOIPs is usually very small, it is only 24 meV in PhE-PbI${}_{4}$ thin film.

In [20], we have noted that the PL intensity of 2DOIPs depends a lot on the nature of the organic and inorganic components. In order to quantify and compare the PL of the different 2DOIPs, we have defined the PL efficiency ρ=I/(P·a), where *I* is the integrated intensity of the PL spectrum, *P* is the power of excitation light, and *a* is the absorption factor at the excitation wavelength (325 nm here). As an example, we were able to demonstrate that the PL efficiencies of the PhE-PbBr${}_{4}$ and (C${}_{6}$H${}_{11}$CH${}_{2}$NH${}_{3}$)${}_{2}$PbBr${}_{4}$ were 3.59 and 7.64 times larger than the PL efficiency of PhE-PbI${}_{4}$, respectively [20].

The 2DOIP nanoparticles show also good PL efficiency. Figure 7(b) and Figure 8(b) present the PL of PhE-PbI${}_{4}$ nanoparticles. The PL spectrum of the PhE-PbI${}_{4}$ 2DOIP nanoparticles is very similar to that from of the 2DOIP thin film. However the PL maximum of the 2DOIP nanoparticles presents a slight (several nanometers) red shift compared to the thin film. Moreover, the FWHM of the PL spectrum of PhE-PbI${}_{4}$ nanoparticles is a bit larger (FWHM = 87 meV) than that of the PhE-PbI${}_{4}$ thin film. Such differences between the PL spectra of the nanoparticles and thin films have also been observed in other 2DOIPs [21].

#### 3.2.4. Low temperature absorption spectroscopy

**Figure 9:**
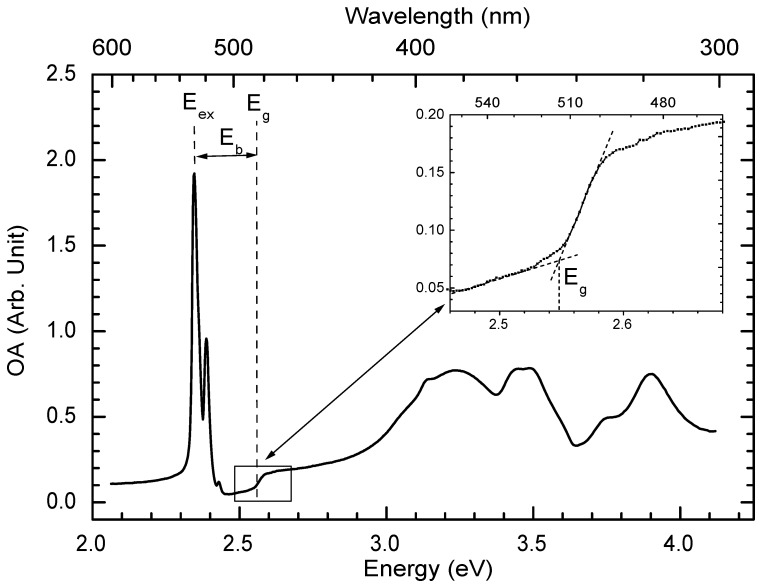
Optical absorbance (OA) spectrum of 50-nm-thick PhE-PbI${}_{4}$ film measured at 10 K. The inset is a zoom of the square zone. E${}_{ex}$, E${}_{g}$, and E${}_{b}$ represent the exciton energy, the enery gap, and the exciton binding energy, respectively.

Figure 9 presents the OA spectrum of a 50-nm-thick PhE-PbI${}_{4}$ film measured at 10 K. The PhE-PbI${}_{4}$ film was prepared by spin-coating 0.1 mol L${}^{-1}$ perovskites in DMF on quartz slide at 2000 rpm for 30 seconds. The low temperature OA spectrum was measured by a home-made experimental setup. Light from a Xenon lamp was coupled through an optical fiber into a monochromator. The monochromatic light was then collimated by a fused silica lens and passed through the sample, which was settled on the cold finger of a cryostat. One quartz slide was introduced into the optical path before the cryostat to reflect a small portion light as a reference. The intensity of the reflected reference light ($I_{r}$) and the intensity of the light passed through the sample ($I_{s}$) were collected by two identical photomultipliers (PMT). Signals of the two PMT were acquired by two lock-in amplifiers, connected to a computer through GPIB interfaces. A clean quartz substrate was firstly installed on the cold finger of the cryostat, and PMT signals of reference light ($I_{r0}$) and transmitted light ($I_{s0}$) were recorded as a function of light wavelength (λ). Then the 2DOIP sample was installed on the cold finger of the cryostat, and PMT signals of the reference light ($I_{rT}$) and transmitted light ($I_{sT}$) were recorded at low temperature. The OA spectrum is obtained by OA(λ) = $-\log_{10}\left(\frac{I_{r0}/I_{s0}}{I_{rT}/I_{sT}}\right)$. Effect of intensity fluctuations of the light source was removed in this two PMT configuration.

Figure 9 shows that the exciton energy in PhE-PbI${}_{4}$ is $E_{ex}$ = 2.346 eV. A step-like structure (as zoomed in Figure 9) is also observed, which corresponds to the energy gap of PhE-PbI${}_{4}$ ($E_{g}$ = 2.548 eV). An exciton binding energy of $E_{b}$ = 202 meV is then deduced for PhE-PbI${}_{4}$. The binding energy of excitons in PbI${}_{2}$ crystals has been determined to be 30 meV [42]. Thus, the exciton binding energy in 2DOIPs is much higher than that in the 3D structures. In fact, theoretical exciton binding energy is found to be four times larger in an ideal 2D structure than in a 3D structure [43]. In addition, by virtue to the high contrast in dielectric constants between the organic layers and the inorganic PbI${}_{4}$ layers, the Coulomb interaction in the well is hardly screened by the presence of the barrier, resulting in a strengthening of the interaction between the electron and the hole in the exciton. Note that the exciton binding energy in 2DOIPs (E${}_{b}\approx$ several hundreds meV) is much larger than the thermal energy at room temperature ($k_{B}T$ = 26 meV), which explains the strong excitonic absorption and PL features observed at the room temperature.

## 4. Influence of the Different Components in (R-NH${}_{3}$)${}_{2}$MX${}_{4}$

### 4.1. Influence of the inorganic part MX${}_{2}$

The absorption wavelength range of the 2DOIPs is related to the nature of the inorganic components. In the 2DOIPs with general chemical structures (R-NH${}_{3}$)${}_{2}$MX${}_{4}$ or (H${}_{3}$N-R-NH${}_{3}$)MX${}_{4}$, M is divalent metal, such as, Pb${}^{2+}$, Sn${}^{2+}$, Ge${}^{2+}$, Cu${}^{2+}$, Ni${}^{2+}$, Mn${}^{2+}$, Fe${}^{2+}$, Co${}^{2+}$, Eu${}^{2+}$, etc. [13,15,16,17], changing M${}^{2+}$ can drastically change the optical properties of the 2DOIPs. For example, the excitonic absorption peaks of PhE-MI${}_{4}$ locate at 2.40 eV (517 nm) for M = Pb [20] and at 2.04 eV (608 nm) for M = Sn [44], the energy of excitonic peak is mainly determined by the electronic transition in the MI${}_{2}$ sheets.

**Figure 10:**
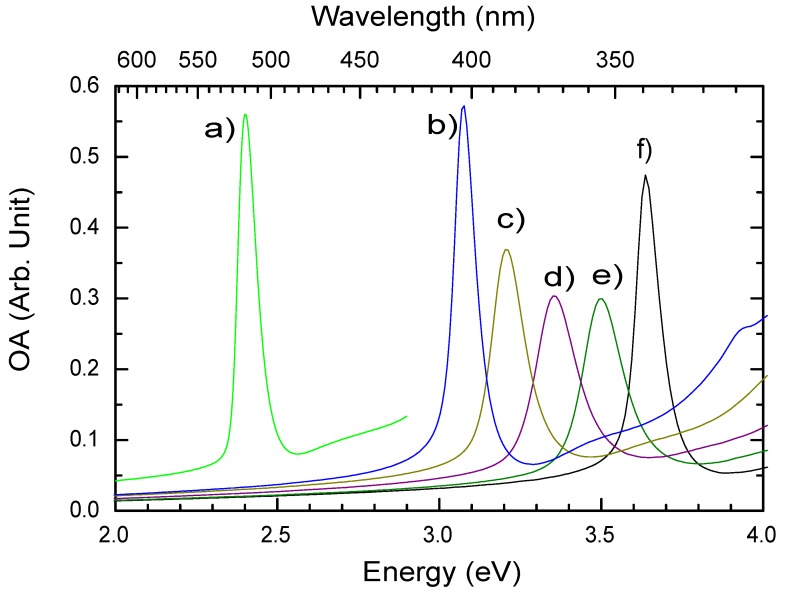
Optical absorbance (OA) spectra of different 2DOIPs measured at room temperature. The chemical formula of each 2DOIP is a) PhE-PbI${}_{4}$, b) PhE-PbBr${}_{4}$, c) PhE-PbBr${}_{3}$Cl, d) PhE-PbBr${}_{2}$Cl${}_{2}$, e) PhE-PbBrCl${}_{3}$, f) PhE-PbCl${}_{4}$, respectively. The 2DOIP films were prepared by spin-coating 0.05 mol L${}^{-1}$ perovskites in DMF on quartz slides at 2000 rpm for 30 seconds. The thicknesses of the 2DOIPs are ∼25 nm.

In a similar way, changing the halogen X can also change the optical properties of the 2DOIPs. Figure 10 presents the OA spectra of PhE-PbX${}_{4}$ for different X. As shown by curves a), b), and f), when X is changed, the excitonic peaks of PhE-PbX${}_{4}$ locate at 2.40 eV, 3.08 eV, and 3.64 eV for X = I, Br, and Cl, respectively. The excitonic absorption peak of PhE-PbI${}_{4}$ is attributed to the electronic transition from Pb(6s)-I(5p) mixed states to Pb(6p) state for PbI${}_{2}$ based 2DOIPs [37]. Similarly, the excitonic absorption peaks are due to the transition from Pb(6s)-Br(4p) states to Pb(6p) state for PbBr${}_{2}$ based 2DOIPs [45], and from Pb(6s)-Cl(3p) states to Pb(6p) state for PbCl${}_{2}$ based 2DOIPs [45].

An interesting property can be found by comparing the curves b–f in Figure 10. Mixing two kinds of halogen into 2DOIPs does not give two excitonic absorption peaks, but one absorption peak whose position depends on the proportion of the two kinds of halogen [45]. It is not necessary that *x* is an integer in the PhE-PbBr${}_{x}$Cl${}_{4-x}$, thus the excitonic absorption energy can be continuously tuned by changing *x* between 0 and 4. The same property is seen in the emission spectra: the PL peak positions of the PhE-PbBr${}_{x}$Cl${}_{4-x}$ 2DOIPs are also continuously changed by changing *x* [45].

### 4.2. Influence of the organic part R

Influence of the nature of the organic group on the optical properties of 2DOIPs has not been completely studied at this moment. The influence of the organic group is usually underestimated because they are generally optically inert due to their large energy gap. However, the organic groups definitely influence the properties of the 2DOIP materials. Changing organic groups may drastically change the properties of the 2DOIPs. Moreover, due to the large variety of structures and energy gaps of the organic groups, they are good candidates to finely tailor the properties of 2DOIPs. For example, the exciton energy of 2DOIPs can be tuned by substituting halogens into the organic cations of alkylamonium [46,47] and of phenethylammonium [48,49,50]. Substituting halogens into organic cations may change the energy gaps of organic groups, change the interaction at organic-inorganic interface of 2DOIPs, and distort the inorganic sheet due to steric effect [46,47,48,49,50]. Varying the substituent position can also change the properties of 2DOIPs [50,51]. When organic groups with small energy gaps (such as organic chromophores and laser dyes) are used, the quantum-wells and barriers may be reversed in the 2DOIPs to have organic wells and inorganic barriers [4,5,52,53,54], and efficient luminescence from organic parts has been observed in these 2DOIPs. Organic groups that have polymerization properties, such as polyacetylene, have been reported as the organic parts in 2DOIPs [55,56,57], providing a very high thermal stability (T-d ≈ 300 °C), and efficient PL.

We have studied the influence of organic groups on the properties of PbX${}_{2}$ (X = Br, and I) based 2DOIPs [20]. In order to take the length between the main organic group and the amine unit into consideration, the studied organic groups R-NH${}_{2}$ are represented by a general formula Y-(CH${}_{2}$)${}_{n}$-NH${}_{2}$, where Y is the main organic group, (CH${}_{2}$)${}_{n}$ is the carbon chain between the main organic group Y and the amine unit -NH${}_{2}$. Several aspects should be considered in the choice of the organic groups to improve properties of the (Y-(CH${}_{2}$)${}_{n}$-NH${}_{3}$)${}_{2}$MX${}_{4}$ 2DOIPs, such as homogeneity, self-assembling ability, and luminescence efficiency.

#### 4.2.1. Length of (CH${}_{2}$)${}_{n}$ spacer

The three dimensional structure of the PhE-PbX${}_{4}$ (X = I, Br, Cl) 2DOIPs is sketched in Figure 1. The ammonium unit of organic groups binds to metal halid layer through hydrogen bonding. Due to the geometrical constraint of the ammonium unit, the three hydrogen bondings of each ammonium unit are generally formed either on one bridging and two terminal halogens (as illustrated in Figure 1) or on two bridging halogens and one terminal halogen [16,17]. Thus the ammonium unit penetrates into the inorganic sheets. If the main organic group Y is too large to fit into the holes within the inorganic sheets, a spacer is needed to take the group Y away from the MX${}_{4}^{2+}$ inorganic sheets. We have used (CH${}_{2}$)${}_{n}$ as the spacer, lengths of *n* = 0,1,2,3 has been experimentally tested for different main organic groups Y, such as phenyl, cyclohexane, and adamantane. The length *n* = 0 was found to be too short, introducing some defects in the 2DOIPs crystals, so that the PL of the corresponding 2DOIPs was very weak at room temperature. When *n* = 1, the 2DOIPs begin to have PL at room temperature when the main organic group Y is phenyl or cyclohexane. So we can conclude that the length of the spacer (CH${}_{2}$)${}_{n}$ should be at least n = 1. The optimum length of the spacer (CH${}_{2}$)${}_{n}$ was found to depend on the main organic group R. The optimum length *n* that provides highest PL efficiency is *n* = 1 for (C${}_{6}$H${}_{11}$(CH${}_{2}$)${}_{n}$NH${}_{3}$)${}_{2}$PbBr${}_{4}$ 2DOIPs, and *n* = 2 for (C${}_{6}$H${}_{5}$(CH${}_{2}$)${}_{n}$NH${}_{3}$)${}_{2}$PbBr${}_{4}$ 2DOIPs. The reason will be discussed later in the following section.

#### 4.2.2. Structure flexibility of R

The optical properties of 2DOIPs are correlated with the detailed structure of the inorganic sheets, such as the bond angle and the bond length of metal halide [48]. The organic parts can influence the optical properties by deforming the MX${}_{6}^{4-}$ octahedron sheets differently. The deformation of the MX${}_{6}^{4-}$ octahedron sheets is determined by the forces applied by the organic parts. On the one hand the organic sheets should be rigid enough to bolster up the inorganic sheets, on the other hand the organic sheets should be sufficiently flexible in order not to deform the inorganic sheets too much. Thus the flexibility of the organic group has to be studied. We have found that the PL efficiency of (C${}_{6}$H${}_{11}$CH${}_{2}$NH${}_{3}$)${}_{2}$PbBr${}_{4}$ is two times higher than that of PhE-PbBr${}_{4}$ [20]. We explained this improvement by the flexibility of the organic groups. The main organic group Y in PhE is phenyl, the strong intramolecular electronic interactions in phenyl make it rather rigid. However, main organic group Y in C${}_{6}$H${}_{11}$CH${}_{2}$NH${}_{3}^{+}$ is cyclohexane, which is relatively more flexible than the phenyl moiety, thus resulting in a better fit with the inorganic sheet.

The flexibility of the organic group Y-(CH${}_{2}$)-NH${}_{3}$ includes the effect of both Y and the spacer. The longer the spacer, the more flexible the organic group. Thus the optimum length of spacer that provides highest PL efficiency was found to be n = 1 for the relatively flexible cyclohexane, and n = 2 for the relatively rigid phenyl [20].

#### 4.2.3. Steric encumbrance of Y

The steric encumbrance or structure of the main organic group Y should be neither too large nor too small. As illustrated in Figure 1, the projection of Y on the inorganic MX${}_{4}$ layer should not exceed the square surface formed by the four adjacent Pb ions, otherwise it will hinder its neighboring main organic groups. Thus it is preferred that the organic groups have a chain like structure, such as alkylamine (C${}_{n}$H${}_{2n+1}$NH${}_{2}$, n = 4, 6, 8, 9, 10, 12 [38], and n = 12, 16, 18 [58]), 2-phenylethanamine, and cyclohexylmethanamine [20]. The organic groups R-NH${}_{3}^{+}$ can not be too small, otherwise they will fit into the hole within the inorganic sheets, resulting in a 3D (R-NH${}_{3}$)MX${}_{3}$ structure instead of the 2D (R-NH${}_{3}$)${}_{2}$MX${}_{4}$ one. Let us use A to present the whole organic group (R-NH${}_{3}^{+}$). The maximum size of A for which the perovskites exist in 3D structure can be obtained from a geometrical calculation:

(8) $$\left(r_{A}+r_{X}\right)=t\sqrt{2}\left(r_{M}+r_{X}\right)$$

where t≈1 is a tolerance factor [17]. The lattice constants of PbX${}_{4}$ inorganic sheets in perovskites were measured to be *a* = 5.901 Å for X = Br and a = 6.3285 Å for X = I [29]. The radii of metal and halogen in perovskites are closer to the ionic radii, which are r${}_{Pb}$ = 1.19 Å , r${}_{I}$ = 2.20 Å, r${}_{Br}$ = 1.96 Å, respectively. Take the ionic radii value, we obtain the maximum radius $r_{A}$ is 2.49 Å for PbBr${}_{2}$ and 2.59 Å for PbI${}_{2}$ based 3D perovskites. Such a small value can only be satisfied by small ammonium salts, such as methanammonium or ions [59].

#### 4.2.4. Interaction between R

In the 2D (R-NH${}_{3}$)${}_{2}$MX${}_{4}$ structures, we have ionic bonds in the ionic MX${}_{4}$ sheets, and hydrogen bond between the organic and inorganic sheets. However, the interaction between the two organic sheets containing alkyl chains [38,58] or cyclohexanes [20] usually consists in weak van der Waals forces. If the interaction between the R-NH${}_{3}$ is too weak, it will be difficult to grow high quality 2DOIP crystals. On the contrary, if the main organic group Y have some unsaturated bonds, such as in aromatic components, the adjacent main organic groups can interact with each other through π-stacking or a similar interaction. The interaction between the organic groups can be consequently increased. It is known that there exists π-π interactions between phenyl groups, as a consequence the homogeneity of the PhE-PbX${}_{4}$ films is better than that of (C${}_{6}$H${}_{11}$CH${}_{2}$NH${}_{3}$)${}_{2}$PbX${}_{4}$ films [20]. Besides the bi-organic layer (R-NH${}_{3}$)${}_{2}$MX${}_{4}$, there also exists other 2DOIPs with general formula (NH${}_{3}$-R-NH${}_{3}$)MX${}_{4}$, interaction between the two ammonium units of each (NH${}_{3}$-R-NH${}_{3}$) results in rather strong covalent bonds, thus (NH${}_{3}$-R-NH${}_{3}$)MX${}_{4}$ films can be expected to have better homogeneity.

## 5. Conclusions

We reviewed different methods of synthesis, structural and optical characterizations of the luminescent two-dimensional organic-inorganic hybrid perovskite semiconductors (R-NH${}_{3}$)${}_{2}$MX${}_{4}$. The preparation methods of 2DOIP thin layers and nanoparticles were introduced in details. We discussed the influence of the organic and inorganic components on the properties of 2DOIPs. Special attention was given to the way of choosing the organic parts in order to improve the optical properties. The length of spacer, steric encumbrance and structure flexibility of the organic group, and interaction between the organic groups, play an important role. In order to use the 2DOIPs in optical devices, some additional studies about their photostability have to be performed. Some optically active organic groups, such as, laser dyes and fluorescent substance may be used as the organic groups of the 2DOIPs. This kind of 2DOIPs may be expected to combine both the advantages of organic and inorganic parts. The 2DOIP semiconductors may be potential materials for realizing new generation compact light sources and nonlinear optical devices.

## Figures and Tables

**Table 1:** Parameters used to fit the refractive index of PhE-PbI${}_{4}$ film with Equation 7 [40].

		$A_{i}$ (eV${}^{2}$)	E${}_{0i}$ (eV)	$\gamma_{i}$ (eV)
$\epsilon_{\infty }$	1.86	-	-	-
$\epsilon_{1}$	-	0.825	2.394	0.041
$\epsilon_{2}$	-	3.73	3.25	0.45
$\epsilon_{3}$	-	38.5	5.1	3.9
